# Evaluation of variability in target volume delineation for newly diagnosed glioblastoma: a multi-institutional study from the Korean Radiation Oncology Group

**DOI:** 10.1186/s13014-015-0439-z

**Published:** 2015-07-02

**Authors:** Chan Woo Wee, Wonmo Sung, Hyun-Cheol Kang, Kwan Ho Cho, Tae Jin Han, Bae-Kwon Jeong, Jae-Uk Jeong, Haeyoung Kim, In Ah Kim, Jin Hee Kim, Sung Hwan Kim, Suzy Kim, Dong Soo Lee, Me Yeon Lee, Do Hoon Lim, Hye Li Park, Chang-Ok Suh, Sang Min Yoon, Il Han Kim

**Affiliations:** Department of Radiation Oncology, Seoul National University College of Medicine, Seoul, Korea; Graduate School of Convergence Science and Technology, Seoul National University, Seoul, Korea; Dongnam Institute of Radiological and Medical Sciences, Busan, Korea; Research Institute and Hospital, National Cancer Center, Gyeonggi-do, Korea; Hallym University Kangdong Sacred Heart Hospital, Seoul, Korea; Gyeongsang National University Hospital, Gyeongsangnam-do, Korea; Chonnam National University Hwasun Hospital, Jeollanam-do, Korea; Hallym University Dongtan Sacred Heart Hospital, Gyeonggi-do, Korea; Seoul National University Bundang Hospital, Gyeonggi-do, Korea; Keimyung University Dongsan Medical Center, Daegu, Korea; St. Vincent’s Hospital, The Catholic University of Korea College of Medicine, Gyeonggi-do, Korea; Seoul National University Boramae Medical Center, Seoul, Korea; Uijeongbu St. Mary’s Hospital, The Catholic University of Korea College of Medicine, Gyeonggi-do, Korea; Hallym University Sacred Heart Hospital, Gyeonggi-do, Korea; Samsung Medical Center, Sungkyunkwan University School of Medicine, Seoul, Korea; Jeonju Jesus Hospital, Jeollabuk-do, Korea; Yonsei University College of Medicine, Seoul, Korea; Asan Medical Center, University of Ulsan College of Medicine, Seoul, Korea; Cancer Research Institute; Institute of Radiation Medicine, Seoul National University College of Medicine, 101 Daehak-ro Jongno-gu, Seoul, 110-744 Korea

**Keywords:** Glioblastoma, Target volume, Peritumoral edema, STAPLE, Margin

## Abstract

**Background:**

This study aimed for a collaborative evaluation of variability in the target volumes for glioblastoma, determined and contoured by different radiotherapy (RT) facilities in Korea.

**Methods:**

Fifteen panels of radiation oncologists from independent institutions contoured the gross target volumes (GTVs) and clinical target volumes (CTVs) for 3-dimensional conformal RT or intensity-modulated RT on each simulation CT images, after scrutinizing the enhanced T1-weighted and T2-weighted-fluid-attenuated inversion recovery MR images of 9 different cases of glioblastoma. Degrees of contouring agreement were analyzed by the kappa statistics. Using the algorithm of simultaneous truth and performance level estimation (STAPLE), GTV_STAPLE_ and CTV_STAPLE_ contours were derived.

**Results:**

Contour agreement was moderate (mean kappa 0.58) among the GTVs and was substantial (mean kappa 0.65) among the CTVs. However, each panels’ GTVs and modification of CTVs regarding anatomical structures varied. Three-fourth of contoured panels’ CTVs encompassed the peritumoral areas of T2-high signal intensity (T2-HSI). Nine of nine GTV_STAPLE_ encompased the surgical cavity and the T1-enhanced lesions. Eight of nine CTV_STAPLE_ encompassed the peritumoral T2-HSI area. The median MARGIN_90_ and the median MARGIN_95_ were 1.4 cm and 1.5 cm, respectively.

**Conclusions:**

Moderate to substantial agreement existed in target volumes for 3-dimensional or intensity-modulated RT determined by radiation oncologists in Korea. According to the estimated consensus contours, the initial CTV encompassed the GTV with margin less than 2.0 cm and the whole peritumoral areas of T2-HSI. The findings of our study propose the need for further studies and modified guidelines.

**Electronic supplementary material:**

The online version of this article (doi:10.1186/s13014-015-0439-z) contains supplementary material, which is available to authorized users.

## Background

The prognosis of glioblastoma multiforme (GBM) has not changed for the past 10 years since the European Organization for Research and Treatment of Cancer (EORTC) and National Cancer Institute of Canada (NCIC) demonstrated a survival benefit with local radiotherapy (RT) plus concomitant and adjuvant temozolomide (TMZ) chemotherapy [1]. In the 1970s and 1980s, whole brain RT was considered optimal. However, multiple series over 20 years including the Brain Tumor Cooperative Group 80–01 randomized trial [2] have established local RT as the current standard. Local RT, with smaller irradiated normal brain volumes, produces less RT-induced neurotoxicity [3, 4]. However, defining the optimal local RT treatment volume in GBM remains controversial.

Local treatment volumes used for GBM have varied among cooperative group trials and large-scale single institution studies, especially differing in margin from the gross target volume (GTV) and inclusion of peritumoral edema. The Radiation Therapy Oncology group (RTOG) recommends that the initial clinical target volume (CTV) encompass the entire T2-high signal intensity (T2-HSI; a mixture of peritumoral edema and infiltrative tumor cells) defined on post-operative magnetic resonance imaging (MRI) plus 2 cm, followed by a boost field defined as the residual T1-enhancement and the surgical cavity plus 2.5 cm (per the RTOG 0525 [5] and RTOG 0825 [6] trials). The rationale for peritumoral edema inclusion is that histologically identified microscopic tumor cells have been found in these areas [7, 8]. In contrast, EORTC recommends that the CTV should encompass the T1-enhancement and surgical cavity plus 2–3 cm without intentional inclusion of the T2-HSI [1]. Reports that the majority of recurrences occur within 2 cm of the primary tumor [9–12] support the EORTC protocols. Furthermore, retrospective studies suggest that the use of a margin less than 2 cm around the GTV or the omission of intentional peritumoral edema inclusion does not alter the failure patterns of GBM [13–15]. Thus, no definite consensus for the RT treatment volume in GBM has been established so far.

For this reason, The Brain Tumor Committee of the Korean Radiation Oncology Group (KROG) initiated the KROG 13–18 study to evaluate current practice and variability of target volume delineation in GBM.

## Methods and materials

### Ethics

This study was performed with the approval of the Health Institutional Review Board of Seoul National University College of Medicine, Seoul, Korea.

### Panels and contouring

Fifteen brain tumor expert radiation oncologists from 15 different institutions in Korea participated in the study. They represented the 37 brain tumor expert radiation oncologists of the brain tumor committee of KROG. Six of the 15 were from large scale institutions treating more than 25 newly diagnosed GBM patients with RT per year. Nine cases representing variable clinical scenarios of GBM were chosen for the study. Clinical information about tumor location, age/sex, extent of surgery, performance status, and pre-RT neurological symptoms was provided (Table 1). Patient history and image sets in ‘Digital Imaging and Communications in Medicine’ formats were provided to the panels. Axial pre- and post-operative MRI, including T1-gadolinium enhanced (T1-GdE) and T2-weighted-fluid-attenuated inversion recovery (T2-FLAIR) images, as well as noncontrast-enhanced axial planning computed tomography (CT) images taken in 3-mm slices composed the image sets. Panels were encouraged to contour the GTV and the initial CTV for all cases within each institution’s planning system with a prerequisite of delivering 3-dimensional conformal RT or intensity-modulated RT (IMRT), since all panels were surveyed to use one of the two modalities in GBM RT. Image fusion of pre-/post-operative MRI and planning CT images were recommended for target contouring. The final contours were returned for central analysis, and for statistical evaluation, they were transferred into the Computational Environment for Radiotherapy Research, version 4.6 (Mathworks, Natick, USA).

**Table 1:** Clinical history of studied cases

Case	Scenario	Age / Sex	Type of surgery^a^	ECOG PS		Pre-RT neurologic symptoms
				Pre-op	Post-op	
1	Post-GTR	M / 23	GTR	1	0	None
2	Extensive peritumoral edema	M / 53	STBx	3	1	Headache and dizziness
3	Multiplicity	M / 56	PR	1	0	None
4	Near-falx location	M / 31	GTR	1	1	Mild headache
5	Midline crossing	F / 74	STBx	3	3	Mood change and helplessness
6	Combined with low grade glioma	M / 42	PR	1	1	Dysphasia, dysarthria, and seizure
7	Infratentorial location	M / 56	STR	2	2	Headache and dizziness
8	Thalamic location	M / 54	STBx	1	1	Right lower extremity weakness
9	Localized ventricular seeding	M / 62	STR	1	0	None

*GTR* gross total resection; *STR* subtotal resectionl; *PR* partial resection; *STBx* stereotactic biopsy; *ECOG PS* Eastern Cooperative Oncology Group Performance Status; *RT* radiotherapy

^a^Type of surgery was determined by the neurosurgeon and the postoperative MRI within 48 h

### Panels’ contour agreement

The individual contours were all merged into a single scan. The apparent agreement and kappa-corrected agreement were used to measure consistency between panels [16].

1. Apparent agreement represents the overlap contour obtained by average agreement probability of a voxel selection by the radiation oncologists. The apparent agreement probability of the *i*^*th*^ voxel is calculated as:
  $$ {p}_i=\frac{{\displaystyle {\sum}_{j=1}^m}{r}_j}{m},\ i=1,\dots,\ n $$
  *r*_*j*_ = Rate by which the *j*^*th*^ panel selects the current voxel; in this case of inter-observer analysis, it is 0 or 1.
  m = Number of panels.
  n = Number of voxels selected by any of the panels
2. Generalized kappa statistics are an inter-observer metric that corrects for agreement that could be obtained by chance, and the kappa value is calculated as follows [17, 18]:
  $$ Kappa = \frac{\left( Apparent\  Agreement - Chance\  Agreement\right)}{\left(1- Chance\  Agreement\right)} $$
  Chance Agreement = the expected agreement by chance alone and is based on marginal totals: ${\displaystyle \prod_{j=1}^m}p\left({r}_j=1\right) + {\displaystyle \prod_{j=1}^m}p\left({r}_j=0\right)$
  The kappa values range between −1 and 1, with a value of −1 representing complete disagreement, 0 representing no agreement above chance, and 1 representing perfect agreement. According to Landis and Koch’s interpretation of strength of agreement, kappa <0.00 is poor, 0.00 − 0.20 is slight, 0.21 − 0.40 is fair, 0.41 − 0.60 is moderate, 0.61 − 0.80 is substantial, and 0.81 − 1.00 is near perfect agreement [19].

### Estimated consensus contours; GTV/CTV_STAPLE_

Consensus generation was done by maximum likelihood estimation using the simultaneous truth and performance level estimation (STAPLE) algorithm. Using the collection of manually drawn contours provided by panels, the STAPLE algorithm computes a probabilistic estimate of the ‘true contour’ that represents the desired tumor and measures the performance of each segmentation [20]. The probabilistic estimated consensus contours of GTV and CTV of each case were generated in the form of GTV_STAPLE_ and CTV_STAPLE_, respectively, at a 95 % confidence level.

1. Margin
  To assess margin the from GTV_STAPLE_ to CTV_STAPLE_, MARGIN_90_ and MARGIN_95_ were used. MARGIN_90_ and MARGIN_95_ were the minimal margins needed to cover at least 90 % and 95 % of the CTV_STAPLE_ volume, respectively. The reason 100 % coverage margin was not used was to allow for the effects of variations in each physician’s policy of contouring regarding clinical scenarios, irregularity of the contour, anatomical modification of the CTVs considering bony or ventricular structures, or inclusion of peritumoral edema within the CTVs.
2. Comparison with RTOG and EORTC
  For comparison of the CTV_STAPLE_ and the CTV based on RTOG/EORTC guidelines, CTV_RTOG_ and CTV_EORTC_ were manually contoured in each case by a single observer. Regarding the CTV_EORTC_ delineation, a 2.5-cm margin was utilized. The dice similarity coefficient (DSC) index was used for comparison of CTV_RTOG_ and CTV_EORTC_ overlap with the CTV_STAPLE_ volume. DSC is calculated as follows:
  $$ \frac{2\ *\ \left[\mathrm{Volume}\ \mathrm{of}\kern0.5em \left({\mathrm{CTV}}_{\mathrm{STAPLE}}\ {\displaystyle \cap}\kern0.5em {\mathrm{CTV}}_{\frac{\mathrm{RTOG}}{\mathrm{EORTC}}}\right)\right]}{\left(\mathrm{Volume}\ \mathrm{of}\kern0.5em {\mathrm{CTV}}_{\mathrm{STAPLE}} + \mathrm{Volume}\ \mathrm{of}\ {\mathrm{CTV}}_{\frac{\mathrm{RTOG}}{\mathrm{EORTC}}}\right)\ } $$
  A DSC of 1 represents perfect overlap and thus perfect agreement whereas the DSC is 0 if no overlap exists [16].

## Results

### Panels’ contour evaluation

**Fig. 1:**
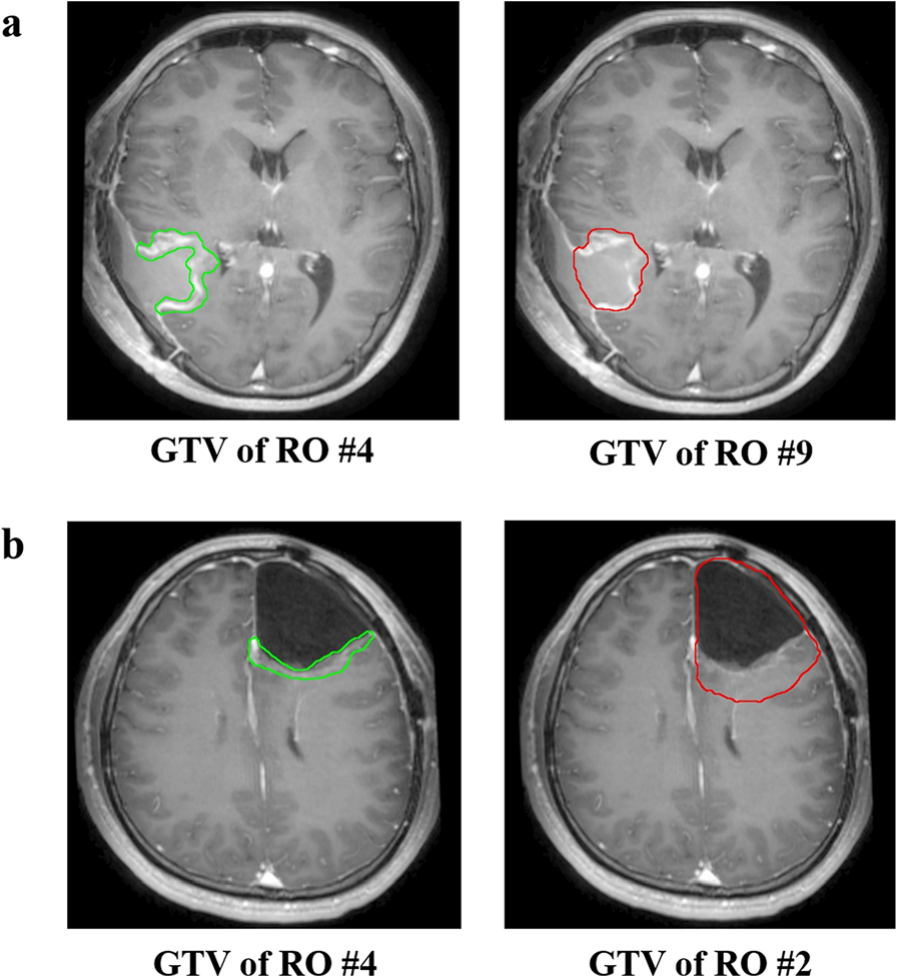
**a** Case 1. RO #4 only included the resection margin for GTV (green line) whereas RO #9 included both the resection margin and the resected tumor bed (red line). **b** Case 4. RO #2 included both the resection margin and the resected tumor bed (red line), in contrast to RO #4 (green line). Abbreviation; GTV, gross target volume; RO, radiation oncologist

All 15 participating panels contoured the GTV and CTV in 9 cases, resulting in a total of 135 GTVs and CTVs each. GTV and CTV delineation reached moderate agreement with mean kappa value of 0.58 and substantial agreement with mean kappa value of 0.65, respectively. The quantitative variability of the panels’ contours and the kappa values of GTV and CTV in each case are shown in Table 2. Of the 135 GTVs, 100 % included the T1-enhancement in the post-operative/biopsy MRI. If surgical resection was performed, the resection margin was included in 100 % of the contours whereas the whole surgical cavity, which indicates the space of post-operative tissue defect with fluid collection created by surgical resection plus the surgical margin, was included in only 81.1 % of the contours. Only 1 radiation oncologist strictly confined the GTV to the resection margin throughout all 9 cases whereas most of the panels included the whole surgical cavity in most cases (Fig. 1). GTV included the T2-HSI in only 34.8 % of the panels’ contours. In contrast, CTV included the whole T2-HSI in 74.8 % (Additional file 1: Table S1).

**Table 2:** Summary of panelist GTV and CTV volume statistics

GTV									
Parameter	Case 1	Case 2	Case 3	Case 4	Case 5	Case 6	Case 7	Case 8	Case 9
Volume maximum (cm^3^)	134.50	123.02	123.06	189.49	284.10	278.52	77.11	146.65	190.20
Volume minimum (cm^3^)	22.34	21.88	34.22	21.61	80.85	18.50	5.13	16.79	10.44
Volume mean (cm^3^)	65.27	43.35	52.07	114.20	106.22	169.86	34.15	61.12	95.24
Volume SD (cm^3^)	±33.42	±27.88	±24.15	±55.31	±50.55	±66.92	±19.84	±36.45	±51.68
Volume SD / volume mean	0.51	0.64	0.46	0.48	0.48	0.39	0.58	0.60	0.54
Volume intersection (cm^3^)	11.59	10.63	19.68	0.84	57.85	13.21	2.34	12.16	6.79
Volume union (cm^3^)	175.36	135.03	162.01	238.47	304.28	327.70	101.84	157.29	256.10
Kappa value	0.58	0.59	0.65	0.51	0.74	0.61	0.49	0.53	0.48
Agreement level	Moderate	Moderate	Substantial	Moderate	Substantial	Substantial	Moderate	Moderate	Moderate
CTV									
Parameter	Case 1	Case 2	Case 3	Case 4	Case 5	Case 6	Case 7	Case 8	Case 9
Volume maximum (cm^3^)	486.35	485.32	531.45	419.49	817.83	564.00	247.17	388.33	636.09
Volume minimum (cm^3^)	61.96	181.74	83.18	194.91	176.76	259.26	64.22	80.23	150.10
Volume mean (cm^3^)	262.42	317.32	303.08	312.48	362.92	410.61	125.78	263.21	371.95
Volume SD (cm^3^)	±104.03	±96.03	±154.41	±84.12	±174.78	±101.84	±56.92	±104.31	±151.42
Volume SD / volume mean	0.40	0.30	0.51	0.27	0.48	0.25	0.45	0.40	0.41
Volume intersection (cm^3^)	25.53	134.41	53.97	46.67	145.25	217.43	31.73	71.49	104.90
Volume union (cm^3^)	572.74	591.32	609.61	534.51	880.28	693.43	311.87	510.41	724.87
Kappa value	0.63	0.71	0.55	0.68	0.64	0.74	0.62	0.62	0.65
Agreement level	Moderate	Substantial	Moderate	Substantial	Substantial	Substantial	Substantial	Substantial	Substantial

*GTV* gross tumor volume; *CTV* clinical tumor volume; *SD* standard deviation

Evaluation of individual radiation oncologist’s modification of CTVs after a simple margin expansion from the GTVs, regarding potential anatomical barriers such as the bony structure, falx, tentorium, and ventricular system was also performed. Constraint regarding the ventricular system means that no portion of the CTV violates into the CSF space of the lateral ventricles, which might occur if the CTV is not modified after simple margin expansion. All radiation oncologists were found to constantly constrain the CTVs in proximity of the bony structures. However, rates of constraining the CTV in regards of the falx, tentorium, ventricular system varied. The falx (80 %) and the tentorium (71 %) were more strictly concerned compared to the ventricular system (34 %). Only 1 radiation oncologist strictly concerned all 3 anatomical barriers throughout all 9 cases. In contrast, 1 radiation oncologist only concerned those 3 barriers in only 22 % of the cases. The details of results are shown elsewhere (Additional file 2: Table S2).

### GTV_STAPLE_ and CTV_STAPLE_

GTV_STAPLE_ included T1-enhancement in the post-operative/biopsy MRI in all cases. In 6 surgically resected cases GTV_STAPLE_ always encompassed not only the resection margin but also the whole surgical cavity. GTV_STAPLE_ and CTV_STAPLE_ included the whole T2-HSI in 2 and 8 cases, respectively. The median MARGIN_90_ and MARGIN_95_ were 1.4 cm (range, 1.0 − 2.5) and 1.5 cm (range, 1.2 − 2.8), respectively. T2-HSI inclusion rates and margin statuses are shown in Table 3.

**Table 3:** T2HSI inclusion rates, MARGIN_90_, and MARGIN_95_ of each case

Case	T2HSI		MARGIN_90_ (cm)	MARGIN_95_ (cm)
	GTV_STAPLE_	CTV_STAPLE_		
Case 1	No	Yes	1.6	1.8
Case 2	No	Yes	1.9	2.2
Case 3	No	Yes	2.5	2.8
Case 4	No	Yes	1.4	1.6
Case 5	No	No	1.1	1.2
Case 6	Yes	Yes	1	1.2
Case 7	No	Yes	1.2	1.4
Case 8	Yes	Yes	1.4	1.5
Case 9	No	Yes	1.9	2.3
Overall	22.20 %	93.30 %	1.4 (median)	1.5 (median)

*T2*-*HSI* T2-high signal intensity; *STBx* stereotactic biopsy

In the comparison between CTV_STAPLE_ and the guideline-based CTVs, the mean volume of the contour was the smallest in CTV_STAPLE_ (329.76 cm^3^) followed by CTV_EORTC_ (349.44 cm^3^ and *CTV*_*R*_*TOG*(458.65*cm*^3^), although the differences were not statistically significant. The mean DSC was 0.77 (range, 0.52 − 0.85) between CTV_STAPLE_ and CTV_RTOG_, and 0.78 (range, 0.69 − 0.86) between CTV_STAPLE_ and CTV_EORTC_ (Table 4). In contrast to CTV_RTOG_, which covers the T2-HSI plus an additional 2 cm, CTV_EORTC_ and CTV_STAPLE_ missed some areas of T2-HSI in 4 and 1 of the 9 cases, respectively.

**Table 4:** Comparison between CTV_STAPLE_, CTV_RTOG_, and CTV_EORTC_

Case	CTV_STAPLE_	CTV_RTOG_		CTV_EORTC_	
	Volume (cm^3^)	Volume (cm^3^)	DSC (with CTV_STAPLE_)	Volume (cm^3^)	DSC (with CTV_STAPLE_)
Case 1	272.04	399.77	0.79	359.65	0.81
Case 2	340.18	447.84	0.84	403.87	0.86
Case 3	377.22	502.03	0.80	365.69	0.83
Case 4	347.00	455.77	0.79	443.21	0.77
Case 5	339.92	945.67	0.52	592.57	0.71
Case 6	424.29	440.22	0.85	304.26	0.79
Case 7	138.82	124.50	0.76	132.26	0.72
Case 8	309.10	251.25	0.77	177.93	0.69
Case 9	419.28	560.82	0.77	365.50	0.84
Mean value	329.76	458.65	0.77	349.44	0.78

*DSC* dice similarity coefficient

## Discussion

RT with or without surgical resection is the standard of GBM treatment. Therefore, a clear definition of the GTV and CTV is necessary. Furthermore, the introduction of TMZ has significantly prolonged the survival in these patients, and reports that specific molecular subgroups survive even longer [1, 21] has made the need for minimizing unnecessary irradiation to the normal brain tissue more urgent. Definite consensus guidelines for target delineation in GBM patients that address this need do not exist.

In this study, moderate and substantial agreements among Korean radiation oncologists were observed for GTV and CTV delineation, respectively. The levels of agreement were comparable to the results of studies involving different diseases that have utilized the same methodology [22–25]. Small heterogeneity might not result in significant differences in actually irradiated volumes of the brain, but because more conformal radiation techniques are available in current practice, accurate target delineation is essential. First of all, accurate delineation of the GTV after surgical resection is important. According to our study, most of the Korean radiation oncologists tended to encompass the whole surgical cavity, which includes both the space of post-operative tissue defect with fluid collection and the resection margin, in the GTVs whereas only 1 radiation oncologist strictly confined the GTVs to the resection margin only in all cases. Fig. 1 shows the difference in 2 cases. Surrounding the brain tissue, there are several dose limiting organs such as the brain stem, spinal cord, optic apparatus, acoustic apparatus, hippocampus, etc. In case 4, in contrast to case 1, the 2 displayed GTVs differ largely in their volumes and the difference is likely to increase when the CTVs are created by a margin expansion. Eventually, at the time of actual planning, the optic apparatus are likely to limit the PTV coverage by dose of 60 Gy or 61.2 Gy at the caudal aspect. Even in case 1, although the volumes of the panels’ GTVs seem not to differ largely, the final CTV or the PTV of the GTV including the whole surgical cavity, if they are created by margin expansion and not constrained to the brain tissue, is likely to end up with a higher dose to the patient’s scalp, resulting in either temporary or permanent hair loss. Constraining the CTV strictly in proximity of the potential anatomical barriers, such as the falx, tentorium, and the ventricular space, is in a similar vein with accurate GTV contouring. Since the space of post-operative tissue defect with fluid collection do not harbor GBM tumor cells, future guidelines should propose a more detailed definition of GTV in GBM. Furthermore, a strict quality assurance for CTV modification regarding the anatomical barriers is necessary, especially in a clinical trial setting.

Numerous data support proximity to the gross tumor as the most important factor in predicting GBM recurrence [9–12]. However, there are caveats to applying these results of older studies to the modern era. Several modern MRI-based studies [15] based on this principle utilized margins less than 2 cm, such as 0.5 cm, 1.0 cm, or 1.5 cm. These studies showed that the centrally failing pattern of GBM does not change even with reduced margins. In our study, the median MARGIN_90_ and MARGIN_95_ were 1.4 cm and 1.5 cm, respectively, which are less than 2 cm or 2.5 cm, as utilized by the RTOG or EORTC. These findings appear to reflect the disregarding of older RTOG/EORTC guidelines by Korean radiation oncologists. The MARGIN_90_ and MARGIN_95_ were 2.5 cm or more only in the case of multicentric GBM (case 3). Multiplicity at initial presentation reflects a tumor of a more infiltrative and aggressive nature compared to tumors presenting as solitary lesions. Demand for highly aggressive therapy with larger RT treatment volumes rather than relying on a single unified guideline may be warranted for these patients. In cases where GTV_STAPLE_ was defined as the T2-HSI (cases 6 and 8) as in the RTOG trials, margins less than 1.5 cm were utilized, reflecting the use of smaller margins than the RTOG guideline in contemporary practice even when T2-HSI is considered the gross tumor. Reduction of treatment volumes based on the evidence of failure patterns would yield smaller RT fields and thus result in lesser neurologic morbidity in longer-surviving patients. However, modern studies [13–15] have not demonstrated the non-inferiority in local control rates. Therefore, to justify the reduced margin below 2 cm, further studies demonstrating equivalent local control with conventional margins are necessary.

**Fig. 2:**
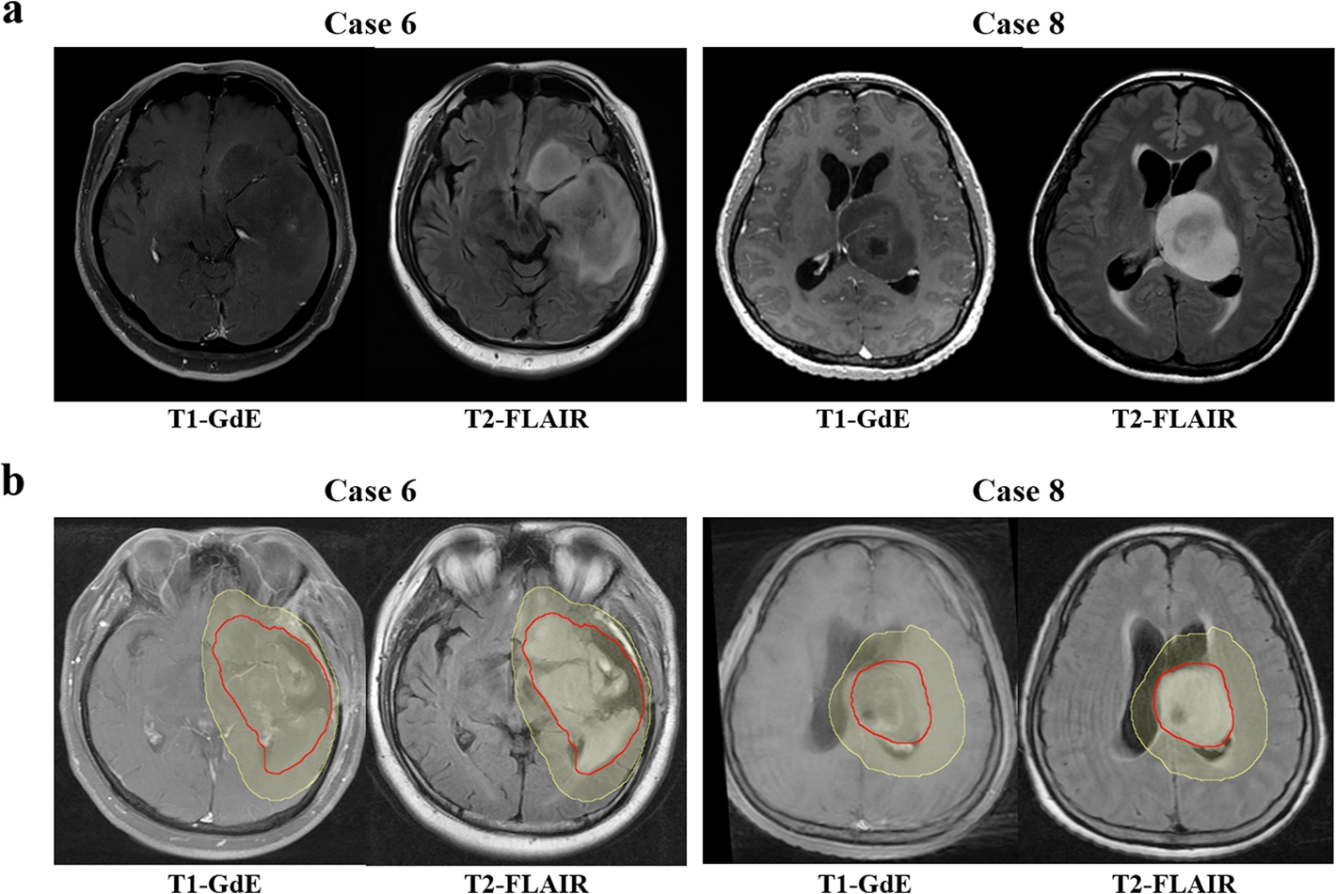
**a** Pre-operative/biopsy gadolinium enhanced T1-weighted (T1-GdE) and T2-FLAIR images of case 6 and 8. **b** GTV_STAPLES_ (red) and CTV_STAPLES_ (yellow) contoured on post-operative/biopsy MRIs of case 6 and 8

Another issue of controversy is whether it is necessary to intentionally include peritumoral edema, which is often simplified as T2-HSI on MRI, within the target volume. Burger *et al*. [7] reported that the hypodense areas surrounding the enhancing lesion on CT images contained infiltrative tumor cells and infiltration of tumor cells may extend even beyond the hypodense areas. Halperin *et al*. [8] also reported that adding 3 cm to the hypodense area is optimal for covering all infiltrative tumor cells. The RTOG protocols mirror these findings. However, modern series utilizing MRI [13–15] indicate that the omission of intentional inclusion of peritumoral edema with reduced margins does not change the failure pattern in GBM. In our study, most panels’ GTVs and GTV_STAPLES_ did not encompass the whole T2-HSI, suggesting that they tend to overlook the RTOG recommendations. The two cases (cases 6 and 8) in which the GTV_STAPLE_ encompassed the whole T2-HSI, images showed mass-like T2-HSI lesions indicating tumor mass rather than edema, whereas areas of enhancement were not definite (Fig. 2). As some portion of GBM patients, up to 10–15 %, present with non-enhancing tumors on MRI, future guidelines should individualize the recommendation based on the initial image findings of the tumor rather than unifying the definition of GTV as the T1-enhancement or T2-HSI. In contrast to the GTV, most of the panels’ CTVs and CTV_STAPLES_ included the whole T2-HSI, indicating that most Korean radiation oncologists do not ignore the possibility of malignant tumor cell existence in the T2-HSI. On the other hand, additional margins around the T2-HSI were not commonly observed in CTV_STAPLES_. Eradicating every malignant tumor cells is impossible, but at the same time, totally neglecting the T2-HSI risks the possibility of marginal recurrence. It would be difficult for the radiation oncologist to ignore an obviously discemible T2-HSI. Moreover, the volumes of CTV_STAPLE_ s encompassing the whole T2-HSI actually did not differ significantly with those of CTV_EORTCS_. One case (case 5) in which the CTV_STAPLE_ missed areas of T2-HSI was a poorly performing patient who underwent stereotactic biopsy without tumor resection. The extensive infiltrative T2-hyperintense and contrast-enhanced lesion involved bilateral frontal lobes, both anterior horns of lateral ventricles, the corpus callosum and bilateral basal ganglias combined with leptomeningeal seeding at the pontomedullary junction (Additional file 3: Figure S1). The volume of CTV_RTOG_ was almost 3 times higher than that of CTV_STAPLE_. The low T2-HSI inclusion rate of 26.7 % in the panels’ CTVs for this case was probably a result of the poor expected overall survival/disease control [26] and the high probability of radiation-related morbidity [3, 4] in case of whole T2-HSI inclusion. Panels may have been reluctant to administer aggressive radical RT in such a case. As for target volumes for other tumors, target volumes of GBM may be modified based on individual clinical settings. In summary, a majority of Korean radiation oncologists include the T2-HSI in the CTV whereas T2-HSI is not routinely included in the GTV unless the T2-HSI lesion forms a mass like appearance with no definitely enhancing lesion.

Several limitations exist in our study. One is that the dose prescription scheme was not acquired. The RTOG recommends 46 Gy in 23 fractions for the initial CTV followed by a boost of 14 Gy in 7 fractions [5, 6], whereas the EORTC recommends 60 Gy in 30 fractions for a single field [1]. Individual institutions may adopt more variable dose fractionation schemes in non-trial based practices [19–21]. Whether the dose is prescribed to the GTV, CTV, or the planning target volume with extra safety-margins may also vary between panels. Lack of information of the RT technique may also weaken our study as the use of IMRT for brain tumors is increasing. The increasing use and importance of functional MRI techniques like spectroscopy and diffuser tensor imaging, or ^11^C-methionine positron emission tomography [27, 28] were also not reflected in the current study. Nevertheless, the key strengths of this study are that the evaluated number of GTVs and CTVs, all contoured by brain tumor expert radiation oncologists, is relatively high [22–25], and that ours is the first report evaluating inter-observer variability in GBM target delineation.

## Conclusions

**Fig. 3:**
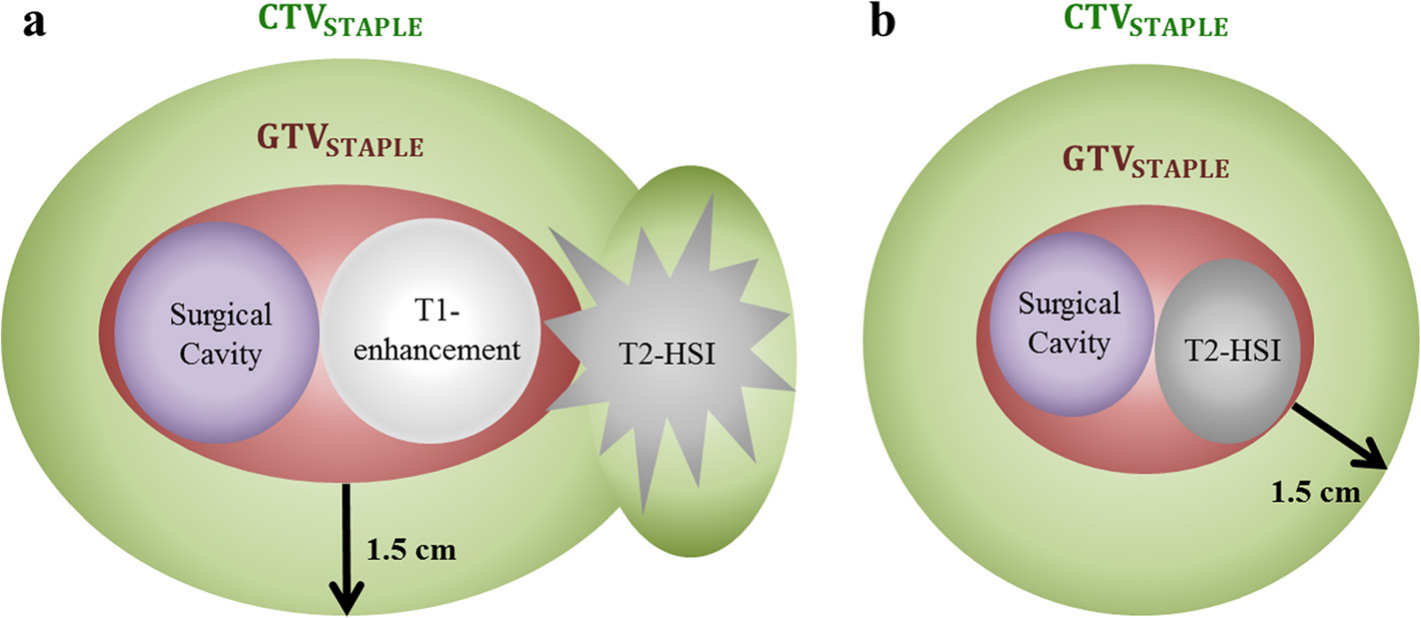
Depiction of the estimated consensus from the Korean Radiation Oncology Group 13–18 study. **a** Enhancing tumors on T1-weighted MRI. **b** Tumors of mass forming T2-HSI without definite enhancement on T1-weighted MRI

Although moderate and substantial agreement were observed between Korean radiation oncologists for GTV and CTV delineation, respectively, several variations were present in delineating the GTV and constraining the CTV in regards of anatomical barriers. Therefore, more detailed guidelines for consistency of target volumes between radiation oncologists are warranted. We found that most of the practicing radiation oncologists tend to define the initial CTV by adding a < 2-cm margin around the GTV and further encompass the remnant T2-HSI uncovered by the margin (Fig. 3), based on modern evidence of failure patterns. In conclusion, these findings of Korean pattern of target volume delineation for GBM propose the need for further studies and modified guidelines of target volume delineation for future clinical trials. Further studies for consensus formation evaluating disease control are ahead from the brain tumor committee of the KROG.

## Additional files

Additional file 1: Table S1. Individual factors included in the GTV and CTV of each case. Additional file 2: Table S2. Modification of CTVs in regards of potential anatomical barriers. Additional file 3: Figure S1. Case 5. a Post-biopsy gadolinium enhanced T1-weighted (T1-GdE) images. b GTV_STAPLE_ (red line) and CTV_STAPLE_ (yellow line) on T2-FLAIR images (areas of T2-high signal intensity uncovered by the CTV_STAPLE_ shown in blue lines).
