# Supplementary material for: Evaluation of variability in target volume delineation for newly diagnosed glioblastoma: a multi-institutional study from the Korean Radiation Oncology Group
Source: Radiat Oncol. 2015 Jul 2;10:137. doi: 10.1186/s13014-015-0439-z (PMC4489390; doi:10.1186/s13014-015-0439-z)
Supplement: Additional file 1: Table S1. — Individual factors included in the GTV and CTV of each case. [file 13014_2015_439_MOESM1_ESM.pdf]

Supplementary 1 Individual factors included in the GTV and CTV of each case

	Surgical cavity[a]	T2-HSI	
Case	GTV	GTV	CTV
Case 1	93.30%	13.30%	80.00%
Case 2	STBx only	20.00%	93.30%
Case 3	100%	6.70%	53.30%
Case 4	73.30%	20.00%	66.70%
Case 5	STBx only	6.70%	26.70%
Case 6	66.70%	86.70%	100%
Case 7	66.70%	86.70%	100%
Case 8	STBx only	60.00%	100%
Case 9	66.70%	6.70%	53.30%
Overall	81.10%	34.80%	74.80%

GTV: gross tumor volume.

CTV: clinical tumor volume.

STBx: stereotactic biopsy.

T2-HSI: T2-high signal intensity.

[a]Surgical cavity is defined as surgical resection margin plus the dead space resulted by brain tissue resection.
