# Supplementary material for: Evaluation of variability in target volume delineation for newly diagnosed glioblastoma: a multi-institutional study from the Korean Radiation Oncology Group
Source: Radiat Oncol. 2015 Jul 2;10:137. doi: 10.1186/s13014-015-0439-z (PMC4489390; doi:10.1186/s13014-015-0439-z)
Supplement: Additional file 2: Table S2. — Modification of CTVs in regards of potential anatomical barriers. [file 13014_2015_439_MOESM2_ESM.pdf]

Supplementary 2 Modification of CTVs in regards of potential anatomical barriers

	Case 1			Case 2			Case 3			Case 4			Case 5			Case 6			Case7			Case8			Case 9			
	F	T	VS	F	T	VS	F	T	VS	F	T	VS	F	T	VS	F	T	VS	F	T	VS	F	T	VS	F	T	VS	Mean
RO 1	1[a]	1	0[b]	1	1	0	1	1	1	1	1	0	1	1	0	1	1	0	1	1	0	1	1	1	1	1	1	0.78
RO 2	1	1	1	1	1	0	1	1	1	1	1	0	1	1	0	1	1	0	1	1	1	0	1	0	1	1	1	0.78
RO 3	1	1	1	1	1	1	1	1	0	1	1	1	1	1	1	1	1	1	1	1	1	0	1	0	1	1	1	0.89
RO 4	1	1	1	1	1	1	1	1	1	1	1	1	1	1	1	1	1	1	1	1	1	1	1	1	1	1	1	1.00
RO 5	1	1	1	1	1	0	1	1	0	1	1	0	1	1	0	1	1	0	1	1	1	0	0	0	1	1	1	0.70
RO 6	1	1	0	0	0	0	1	1	0	1	1	1	1	1	0	1	1	0	0	1	0	0	0	0	1	1	1	0.56
RO 7	0	0	0	0	0	0	0	1	0	0	0	0	1	1	0	0	0	0	0	0	0	0	0	0	1	0	0	0.15
RO 8	1	1	0	1	1	0	1	1	1	0	0	0	1	1	0	1	0	1	1	1	0	0	0	0	1	1	1	0.59
RO 9	1	1	1	1	1	1	1	1	1	1	1	0	1	1	0	1	1	1	1	1	1	0	0	0	1	1	1	0.81
RO 10	1	1	1	1	0	0	1	1	0	0	0	0	1	1	0	1	1	0	1	0	1	0	0	0	1	0	0	0.48
RO 11	1	1	0	1	1	0	1	1	0	1	1	0	1	1	0	1	1	0	1	1	0	1	0	0	1	1	0	0.63
RO 12	1	1	0	1	1	0	1	1	0	1	1	1	1	1	0	1	1	0	1	1	0	1	1	1	1	1	0	0.74
RO 13	1	1	0	0	0	0	0	1	0	0	0	0	1	1	0	0	0	0	0	0	0	0	0	0	0	1	0	0.22
RO 14	1	1	1	1	1	1	1	1	0	0	0	0	1	1	0	1	1	0	1	1	1	0	0	0	1	1	0	0.63
RO 15	1	0	0	1	0	0	1	1	0	0	0	0	0	0	0	1	0	0	1	0	0	0	0	0	1	0	0	0.27
Mean	0.93	0.87	0.47	0.80	0.67	0.27	0.87	0.80	0.33	0.60	0.60	0.27	0.93	0.93	0.13	0.87	0.73	0.27	0.80	0.73	0.47	0.27	0.33	0.20	0.93	0.80	0.53	

Abbreviations; RO, radiation oncologist; F, falx; T, tentorium; VS, ventricular space.

[a]modified; [b]not modified.
