# Supplementary material for: Evaluation of variability in target volume delineation for newly diagnosed glioblastoma: a multi-institutional study from the Korean Radiation Oncology Group
Source: Radiat Oncol. 2015 Jul 2;10:137. doi: 10.1186/s13014-015-0439-z (PMC4489390; doi:10.1186/s13014-015-0439-z)
Supplement: Additional file 3: Figure S1. — Case 5. a Post-biopsy gadolinium enhanced T1-weighted (T1-GdE) images. b GTVSTAPLE (red line) and CTVSTAPLE (yellow line) on T2-FLAIR images (areas of T2-high signal intensity uncovered by the CTVSTAPLE shown in blue lines). [file 13014_2015_439_MOESM3_ESM.pdf]

(a) T1-GdE

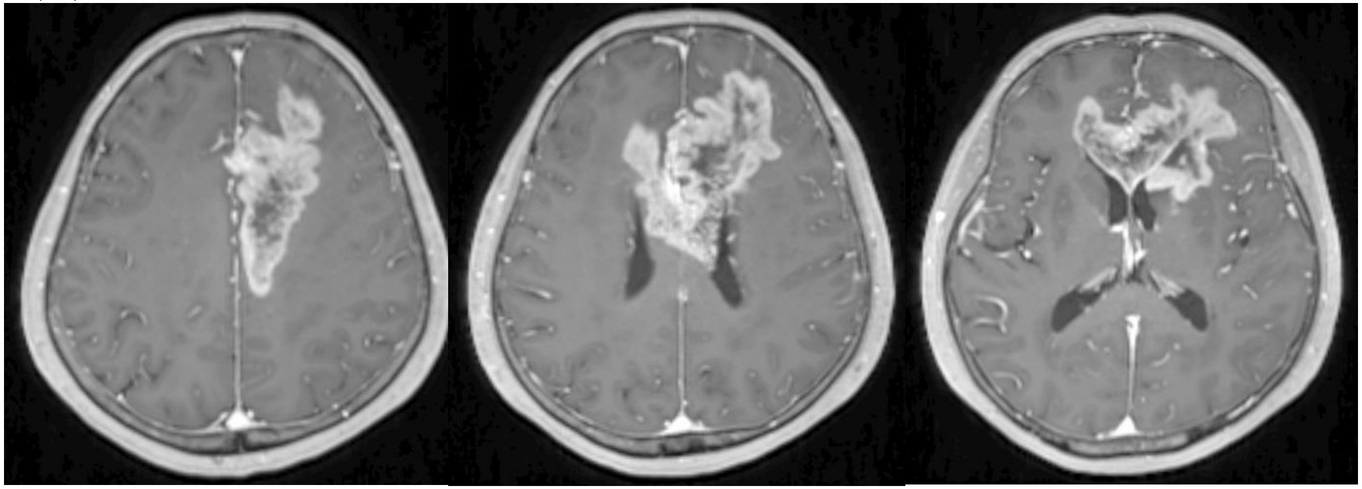

(b) T2-FLAIR

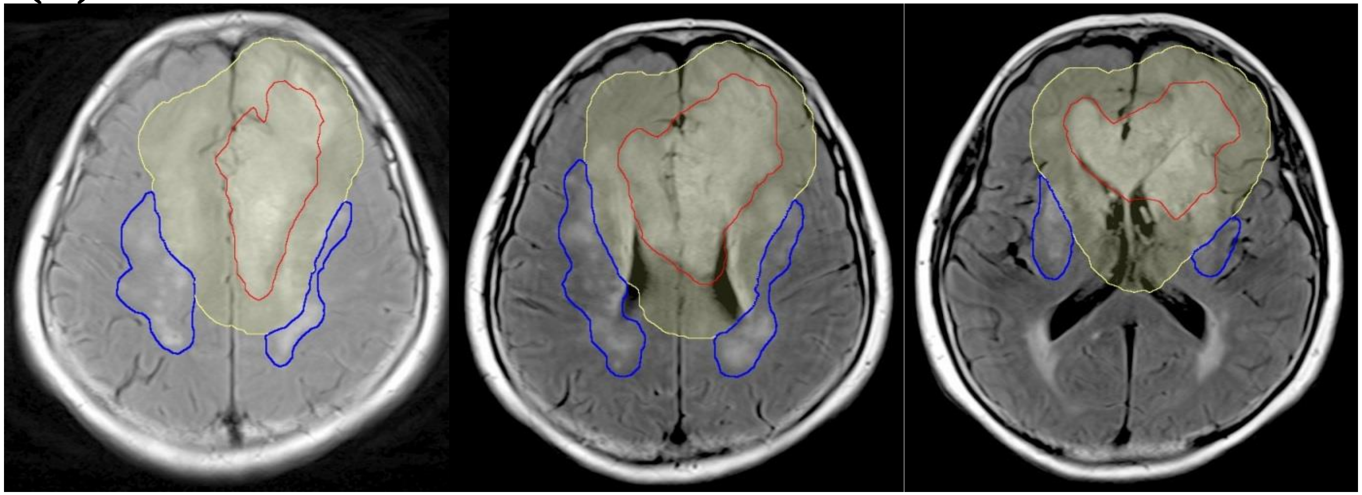

Supplementary 3 Case 5. (a) Post-biopsy gadolinium enhanced T1-weighted (T1-GdE) images. (b) $\mathrm{GTV}_{\mathrm{STAPLE}}$ (red) and $\mathrm{CTV}_{\mathrm{STAPLE}}$ (yellow) on T2-FLAIR images (areas of T2-high signal intensity uncovered by the $\mathrm{CTV}_{\mathrm{STAPLE}}$ shown in blue lines).
